# Pyrolysis of Pruning Residues from Various Types of Orchards and Pretreatment for Energetic Use of Biochar

**DOI:** 10.3390/ma14112969

**Published:** 2021-05-31

**Authors:** Paweł Kazimierski, Paulina Hercel, Tomasz Suchocki, Jakub Smoliński, Agnieszka Pladzyk, Dariusz Kardaś, Justyna Łuczak, Katarzyna Januszewicz

**Affiliations:** 1 Institute of Fluid Flow Machinery, Polish Academy of Sciences, Fiszera 14, 80-231 Gdańsk, Poland; pkazimierski@imp.gda.pl (P.K.); phercel@imp.gda.pl (P.H.); tsuchocki@imp.gda.pl (T.S.); s173393@student.pg.edu.pl (J.S.); dk@imp.gda.pl (D.K.); 2 Department of Energy Conversion and Storage, Chemical Faculty, Gdańsk University of Technology, Narutowicza 11/12, 80-233 Gdańsk, Poland; 3 Department of Inorganic Chemistry, Chemical Faculty, Gdańsk University of Technology, Narutowicza 11/12, 80-233 Gdańsk, Poland; agnieszka.pladzyk@pg.edu.pl; 4 Department of Process Engineering and Chemical Technology, Chemical Faculty, Gdańsk University of Technology, Narutowicza 11/12, 80-233 Gdańsk, Poland; justyna.luczak@pg.edu.pl

**Keywords:** orchard waste, fruit industry waste, biomass pyrolysis, biomass, wood, apple tree, pear tree, plum tree, orchard prunings

## Abstract

The routine pruning and cutting of fruit trees provides a considerable amount of biowaste each year. This lignocellulosic biomass, mainly in the form of branches, trunks, rootstocks, and leaves, is a potential high-quality fuel, yet often is treated as waste. The results of a feasibility study on biochar production by pyrolysis of residues from orchard pruning were presented. Three types of biomass waste were selected as raw materials and were obtained from the most common fruit trees in Poland: apple (AP), pear (PR), and plum (PL) tree prunings. Two heating rates and three final pyrolysis temperatures were applied. For the slow (SP) and fast pyrolysis (FP) processes, the heating rates were 15 °C/min and 100 °C/min, respectively. The samples were heated from 25 °C up to 400, 500, and 600 °C. Chemical analyses of the raw materials were conducted, and the pyrolysis product yields were determined. A significant rise of higher heating value (HHV) was observed for the solid pyrolysis products, from approximately 23.45 MJ/kg for raw materials up to approximately 29.52 MJ/kg for pyrolysis products at 400 °C, and 30.53 MJ/kg for pyrolysis products at 600 °C. Higher carbon content was observed for materials obtained by fast pyrolysis conducted at higher temperatures.

## 1. Introduction

The increase in global energy consumption forces the search for alternative energy sources. In this context, waste biomass produced by the local agriculture industry in various forms can be considered as a valuable source of energy [1,2,3], which additionally may contribute to reducing the environmental impact from emissions and air pollution. The considerations of the possibility of obtaining biochar with a high energy value are useful in the context of broadly understood waste management [4,5,6]. In 2017, they covered an area of approximately 6 million ha in the EU [7], with the most widespread being olive plantations, which occupy 4.5 million ha and are mainly located in Spain and Italy. The largest production area of apple orchards in the EU is located in Poland, occupying approximately 31.1% of the total area of apple orchards [8], whereas pear plantations cover 100,000 ha in the EU and are mainly located in Italy. Since the orchards require regular and annual pruning, cutting, and care, a large amount of wood waste is generated every year [9]. The residual dry biomass from olive annual pruning is about 1.31 t/ha [10], while almond orchards provide 1.34 t/ha [11], and vineyards 4.2 t/ha [12]. This potential high-quality biomass fuel in the form of branches, trunks, rootstocks, and leaves is simply left in the orchard, mulched with energy loss [13,14] instead of being treated as a source of energy. The higher heating value (HHV) of pruned apple residues estimated by Dyjakon [15] was 19.31 MJ/kg of dry mass. The HH values reported by Irawati et al. [16], in turn, were lower; namely, 9.2 MJ/kg for apple residues and 14.6 MJ/kg for plum. In Poland, being an example of the largest apple fruit manufacturer in Europe, the apple orchards occupy over 160,000 ha of the approximately 350,000 ha of all Poland’s orchards [7,17]. Therefore, considering that approximately 3.5 tons per year of biomass can be obtained from 1 ha of fruit tree orchard (2850 trees/ha) [15], it is worth considering that waste as a valuable, abundant fuel.

Some ideas for managing the residues from pruning orchards have been proposed. A three-year experiment conducted by Zhang et al. showed that a compost made from pear tree pruning residue enriched the soil in mineral components with higher efficiency than the chemical fertilizer [18]. Suddick et al. showed that the biochar derived from agricultural wastes amended with soil improved exchangeable ion retention and soil carbon sequestration [5]. Olive and pine pruning were used for the synthesis of geopolymers by Bonet-Martinez et al. [19], who showed that pruning’s waste as raw material can be used in the production of unconventional cement with a 28-day curing strength greater than 10 MPa and thermal conductivity less than 0.35 W/mK. Hoffmann et al. investigated the production of electrochemical capacitors and fuel cells made of conductive materials derived from the hydrothermal carbonization of vineyard residues; however, this area of investigation is still in the laboratory research phase [20].

Taking into account energetic applications, the agricultural biomass, such as olive prunings, cotton residues, olive and peach kernels, pine needles, etc., were combusted as the simplest way to regain energy [21]. Brand et al. [22] showed that in comparison with pine wood residues, a higher amount of apple pruning is required for the generation of one unit of energy (MJ) with a simultaneous lower amount of ash. The authors also demonstrated that the mixture of apple pruning with pine residue produced pellets with higher energy density. Increasing the air–fuel ratio from 1.3 to 1.7 during combustion of orange tree prunings in a laboratory-scale fluidized bed reactor resulted in a decrease of the CO concentration in flue gas from 1600 mg/(Nm^3^) to 700 mg/(Nm^3^), whereas NOx concentration fluctuated at approximately 400 mg/(Nm^3^) [23]. Application of a conical spouted bed for the combustion of fruit tree prunings allowed an increase in the efficiency of the process at lower temperatures, with simultaneous reduction of the amount of generated volatile organic compounds (VOC) generated in comparison with the absence of inert bed [24].

An alternative to combustion is the pyrolysis process, which reduces the content of the gaseous components, and thus the weight and volume of the waste, providing smokeless charcoal. Experimental investigation of pyrolysis of different lignocellulosic biomass types, including olive tree prunings, was conducted by Zabaniotou et al. [25]. The authors showed that the carbon product obtained from the catalytic pyrolysis of olive tree pruning at 500 °C in a fixed bed reactor contained 63.01% carbon, 2.79% hydrogen, and 34.2% oxygen. The lower heating value (LHV) of olive prunings was higher (19.86 MJ/kg) than the other lignocellulosic biomass. Ayala-Cortés et al. [26] performed pyrolysis of agave and tomato prunings at 600 °C and a heating rate of 30 °C/min, obtaining biochar with a carbon content of 60.4%, hydrogen 1.5%, oxygen 29.9%, and sulfur 8.05%. The carbon content in the product obtained at 450 °C was similar (59.8%). Interestingly, Bartoli et al. [27] proposed a biochar production procedure from olive pruning residue using low-temperature microwave pyrolysis, obtaining a relatively large amount of biochar (up to 61.2% product yield).

A literature review showed that studies on pyrolysis of orchard wastes were mainly concerned on olive trees [28,29,30,31] and vineyard pruning residues [32,33,34,35]. Because of the insufficient amount of data on pruning residues from the main orchard trees cultivated in the territory of our country (Poland), we focused our study on the production and analysis of the biochars obtained from pruning residues of apple, pear, and plum. A comparison of the composition of products obtained from slow (15 °C/min) and fast (100 °C/min) pyrolysis experiments conducted at 400, 500, and 600 °C is presented here. The preliminary studies of pyrolysis kinetics and calculation of activation energy of the pyrolysis process were also performed, and are discussed.

## 2. Materials and Methods

### 2.1. Materials and Preliminary Preparation of Samples

**Figure 1:**
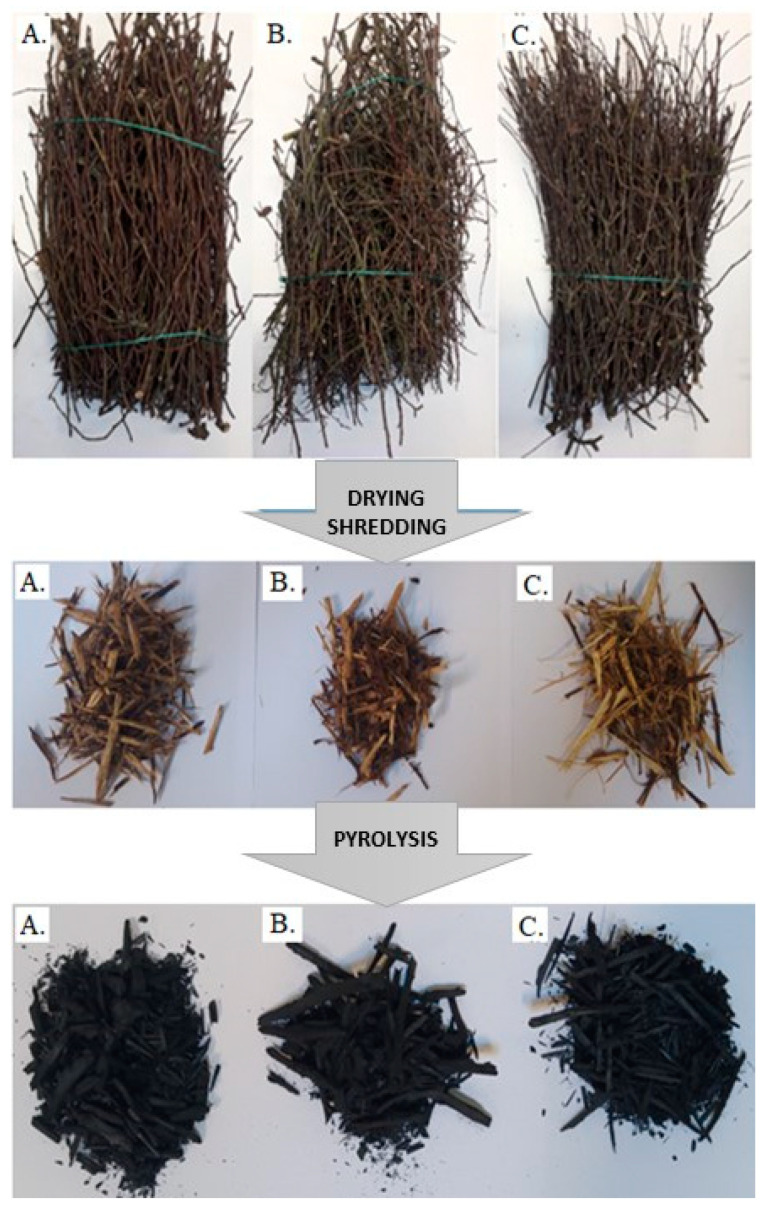
Samples of plum tree (**A**), pear tree (**B**), and apple tree (**C**) prunings as received, prepared for pyrolysis and after pyrolysis.

In this work, the annual pruning residues from home orchards in Poland (Pomerania) were pyrolyzed and characterized. Apple (*Malus domestica*) (AP), pear (*Pyrus communis* L.) (PR), and plum (*Prunus domestica* L.) (PL) trees were chosen due to their widespread occurrence. In the first step, pruning residues as the raw material were cut into wood chips (3–7 cm pieces) using a chipper and then dried at 80 °C for 12 h (Figure 1).

### 2.2. Pyrolysis Process

The pyrolysis process was conducted in an electric muffle furnace (LIFT3.0 + KXP4 R, Neoterm, Wrocław, Poland). Before each experiment, the pyrolysis reactor was purged with nitrogen (inert gas) and then closed. Each biomass sample (approximately 20 g) was thermally treated in a 100 mL steel reactor. Two heating models (15 °C/min and 100 °C/min) and three different final temperatures of the process were applied in the experiments to compare the effects of slow and fast pyrolysis (SP and FP). During the fast pyrolysis process, the steel reactor containing the raw material was placed into a hot furnace heated up to 400, 500, and 600 °C to set specific conditions. After experiments, the biochar samples were cooled to room temperature and removed from the furnace.

### 2.3. Proximate and Elemental Analysis

The ash content of the raw materials was determined according to the Polish standard [36]. The moisture content was analyzed with a MAC moisture analyser (Radwag, Radom, Poland) [37,38]. The elemental analysis of the raw (dried and milled) biomass samples was performed by using the CHNS-O Flash 2000 elemental analyzer (Thermo Scientific, Waltham, MA, USA). The thermogravimetric analysis (TGA) was conducted for the raw materials with a heating rate of 15 °C/min using a TG 209 F3 Tarsus thermo-microbalance (Netzsch, Selb, Germany).

Data obtained from elemental analysis were applied in the calculation of the high heating value (HHV) with the use of the following formula [39]:

HHV = 349.4C + 1178H + 15.1N + 100.5S − 103.4O − 21.1 A

where HHV represents the high heating value; and C, H, N, S, O, and A represent carbon, hydrogen, oxygen, nitrogen, sulphur, and the ash content in the analysed material, respectively.

## 3. Results and Discussion

### 3.1. Characteristics of the Raw Materials

The results of the proximate analysis of pruning residues obtained from apple, pear, and plum trees are presented in Table 1. The moisture content of the analyzed orchard prunings was in the range of 40.91–44.86 wt %, and the highest value was obtained for AP. The higher heating values also did not vary significantly among the analyzed tree species (22.39 and 24.90 MJ/kg), analogous to the ash content (1.33–2.42%).

**Figure 2:**
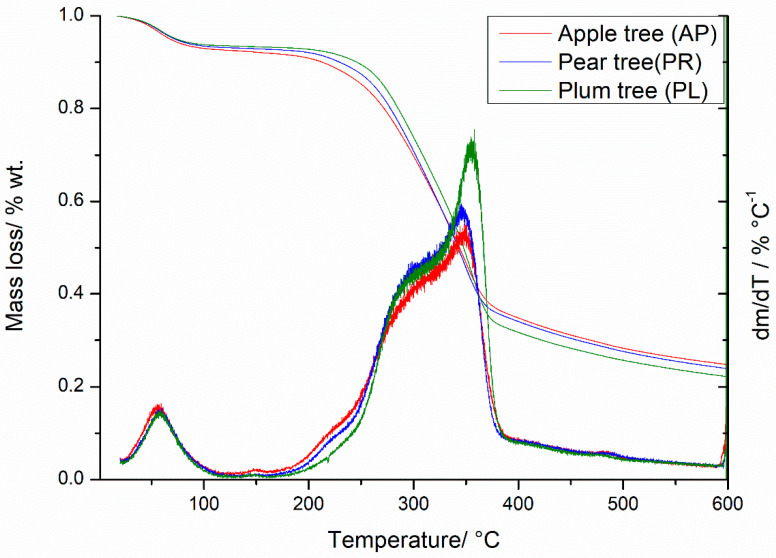
Thermogravimetric analysis of the orchard pruning residues.

The orchards’ pruning wastes were composed of typical wood components like hemicellulose, cellulose, and lignin. To estimate the thermal stability and the pathway of thermal decomposition of the analyzed materials, a thermogravimetric analysis was performed, the results of which are presented in Figure 2. The shapes of the curves reflect the thermal degradation of the biomass in the absence of oxygen. The results obtained during this slow pyrolysis process (the heating rate was 15 °C/min) showed that the degradation of the material was initiated above 110 °C by the loss of water (vaporization). This step reflected the pyrolysis of wet biomass, which is a time- and energy-consuming process, and could be conducted separately on an industry scale [40]. The main degradation step started from about 250 °C up to 390 °C, with the formation of the solid residue in an amount approximately 30% of the initial mass for each sample. Further heating up to 600 °C caused a decrease of this mass by approximately 10%. At this stage, the volatile pyrolysis residues trapped in the pores were removed, and the degradation of lignin was completed. The derivative mass loss curves (dm/dt) present characteristic peaks for the biomass components with hemicellulose degradation in the range of 250–350 °C, cellulose 280–380 °C, and lignin 300–450 °C [41]. The higher peak with a maximum at 355 °C detected for PL reflected the higher cellulose content in the plum tree waste in comparison with the other tree samples.

The comparison of the above-described results with wooden biomass revealed the presence of a twofold process with overlapping stages [42]. The mass loss curve was more inclined in the main decomposition temperature range, and the values of dm/dt were higher. These differences may be related to the higher bark content in the orchard prunings.

### 3.2. Pseudo-Activation Energy of the Pyrolysis

The pseudo-activation energy of the pyrolysis process can be calculated by using the model fitting method [43], which includes the reaction order (*n*) and nonisothermal kinetic parameters for solid fuel pyrolysis to determine the reaction rate (*dx*/*dt*) according to the Arrhenius equation, Equation (1):

(1) $$\frac{dx}{dt}=k{\left(1-x\right)}^{n}$$

where *t* is time (s), *x* is the conversion fraction of fuel sample (1), and *k* is the rate constant (1/s) given by Equation (2):

(2) $$k=Ae^{-\frac{E}{RT}}$$

where *A* is the pre-exponential factor, *E* is the activation energy (J/mol), *T* is the temperature (°C), and *R* is the gas constant.

The conversion fraction of the solid sample in the process was calculated as a function of the current mass, as defined by Equation (3):

(3) $$x=\frac{m_{0\;}-m}{m_{0\;}-m_{\infty }}$$

where *m*_0_ is the initial mass of the sample (g), m is the mass at time t, and *m*_∞_ is the mass at the final temperature.

For a constant heating rate ($\beta =\frac{dT}{dt}$) and first-order kinetic reaction (n = 1), by which the pyrolysis process is described, Equations (1) and (2) can be integrated and rearranged into the Redfeld and Coast Equation (4). This approach was used to calculate the activation energy of the pruning residues pyrolysis process:

(4) $$\ln(-\ln\frac{\left(1-x\right)}{T^{2}})=\ln\frac{AR}{\beta E}\left(1-\frac{2RT}{E}\right)-\frac{E}{RT}$$

where *A* is the pre-exponential factor and *R* is the gas constant (J/molK).

Equation (4), after approximate integration by the simplification of the value of *2RT*/*E*, which is low for most reactions (*2RT*/*E* << 1), gives Equation (5):

(5) $$\ln(-\ln\frac{\left(1-x\right)}{T^{2}}\;)=\ln\frac{AR}{\beta E}-\frac{E}{RT}$$

**Figure 3:**
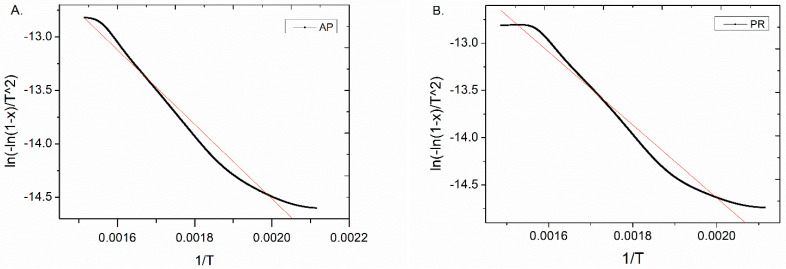

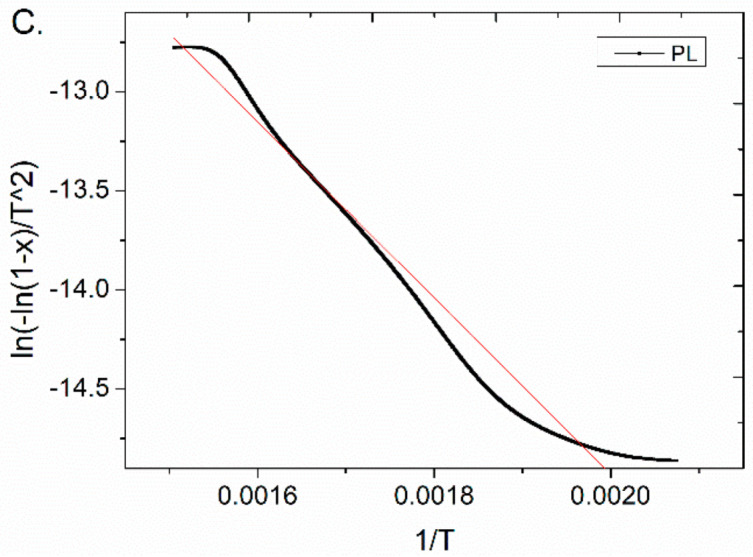
Arrhenius plots for the pyrolysis process of (**A**) apple (AP), (**B**) pear (PR), and (**C**) plum (PL) prunings.

The kinetic analysis of the pyrolytic degradation process was carried out in the temperature range of 200 to 400 °C, and the initial and final sample masses were calculated in this range. This temperature range reflects the conversion of 10–60 wt % of biomass. The results of the TGA performed for AP, PR, and PL recalculated by using Equation (5) are presented in Figure 3.

The pseudo-activation energy obtained from the slope (−*E*/*R*) and correlation coefficient determined for the pyrolysis of the selected orchard residues are summarized in Table 2.

The pseudo-activation energy of the thermal decomposition of prunings performed at a heating rate of 15 °C/min was found to be 28.97 kJ/mol for AP, 32.08 kJ/mol for PR, and 37.00 kJ/mol for PL. The activation energy and the TGA results depended on the amount of components in the biomass structure [41]. Moreover, the activation energy was closely related to the kinetics of chemical reactions occurring during pyrolysis, and the highest amount of energy needed to perform pyrolysis of PL (37 kJ/mol) may be due to the highest dynamics of the process revealed by the TGA (Figure 2). The highest peak of the derivative curve (dm/dT) at 355 °C reflected cellulose degradation (Figure 2). Based on these observations, we assumed that the degradation of cellulose was the rate determining step of the whole orchard pruning pyrolysis process.

### 3.3. Pyrolysis Process of Orchard Residues

**Figure 4:**
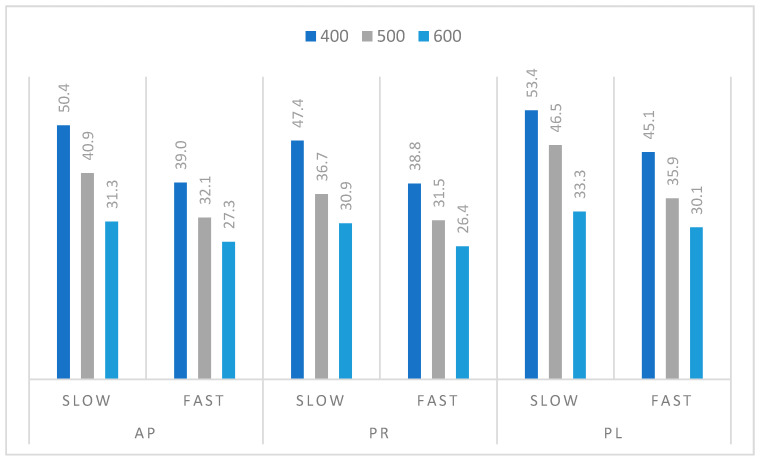
Biochar yield (wt %) from pyrolysis of residual biomass from tree prunings from orchards carried out at different temperatures and heating rates (slow pyrolysis—15 °C/min and fast pyrolysis—100 °C/min).

The yields of the solid residues received as a result of the slow (15 °C/min) and fast (100 °C/min) pyrolysis of the residual biomass from tree prunings from orchards, performed in a 100 mL reactor, are presented in Figure 4. The increasing temperature of the process in the range of 400–600 °C resulted in a decrease in the yield of the biochar produced (e.g., for AP: 50.4 wt % at 400 °C, 40.9 wt % at 500 °C, and 31.3 wt % at 600 °C). A higher temperature of the thermal treatment facilitated the release of the volatile fractions and higher efficiency of the biomass degradation, resulting in a lower biochar yield. Comparison of the slow and fast heating rates indicated the lower amount of solid fraction formed during FP. During SP, the dynamics of the biomass degradation was reduced, and secondary oxidation and possible combustion processes may have occurred [44]. The combination of both parameters; namely, higher heating rate and higher temperature of the pyrolysis, resulted in increased volatile fraction generation, and thus a lower biochar yield. In this regard, the highest biochar yields were obtained during SP performed at 400 °C; namely, 53.4 wt % for PL, 50.4 wt % for AP, and 47.4 wt % for PR. The lowest biochar yield, in turn, was produced during FP of PR performed at 600 °C (26.5 wt %), followed by AP (27.3 wt %) and PL (30.1 wt %). An analogous relation also was observed by other authors who described various wood biomasses such as birch wood [45], pine wood [46], or other wood-based materials [42].

**Figure 5:**
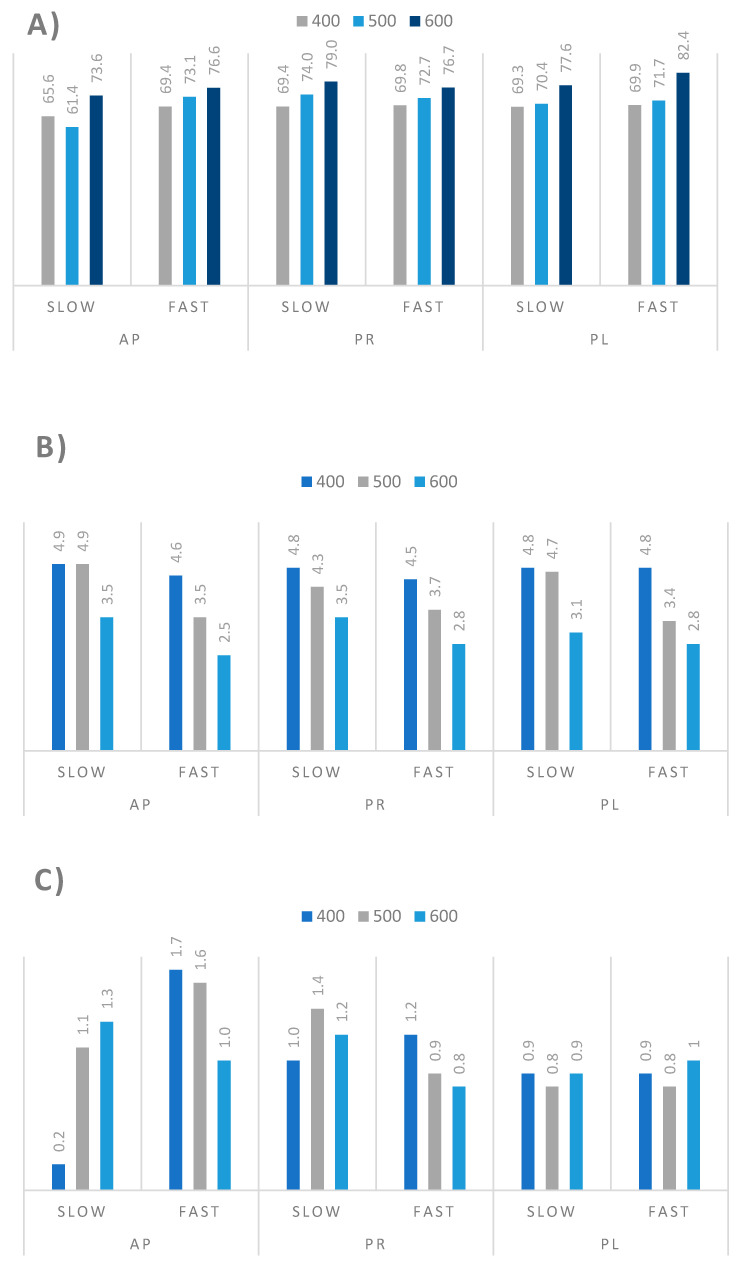
Elemental analysis of the biochar samples produced by the pyrolysis of the residual biomass from tree prunings from orchards: (**A**) carbon content (wt %), (**B**) hydrogen content (wt %), (**C**) nitrogen content (wt %).

Elemental composition (carbon, hydrogen, and nitrogen content) of the biochar samples was compared, and is presented in Figure 5. Disregarding the heating rate, the higher temperature of the pyrolysis process provided the product containing more carbon and less hydrogen [47]. The heating rate did not significantly affect the carbon content in the biochar samples, whereas the nitrogen content in all analyzed samples was low, about 1 wt %.

**Figure 6:**
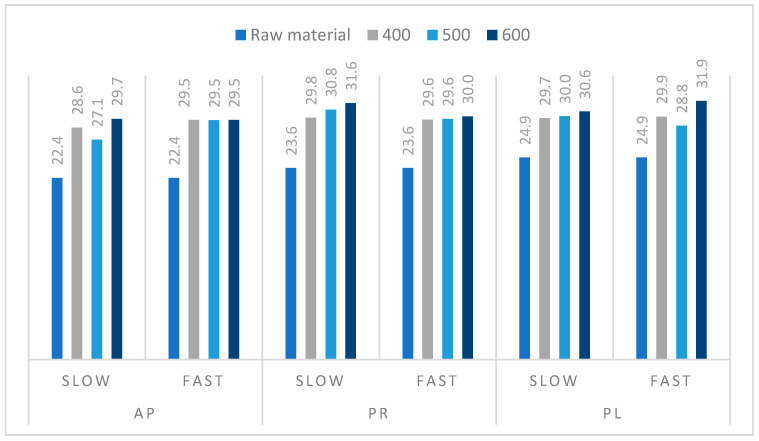
Comparison of higher heating values (MJ/kg) of the raw materials and biochar samples obtained during fast and slow pyrolysis processes.

To verify the potential usability of the obtained biochar as an energy source, the higher heating values were determined, and the results are presented in Figure 6. The highest HHV was achieved for PR pyrolyzed at 600 °C by SP (31.6 MJ/kg) and for PL produced at 600 °C by FP (31.9 MJ/kg). These results indicated that the heating rate of the process had no significant influence on the amount of heat released during the combustion at specific conditions. On the other hand, the highest difference (and thus increment) between HHVs of the biochar and the raw material, 7.3 MJ/kg, was detected for AP processed by SP carried out at 600 °C. These differential values were relatively lower when compared with those received for RDF (18.0–33.0 MJ/kg) [48,49] or tires/rubber wastes (28.0–40.0 MJ/kg) [48,50,51]. However, taking into account the fact that the tested biomass forms abundant and renewable waste, the orchard prunings still may be considered as a valuable source of energy.

**Figure 7:**
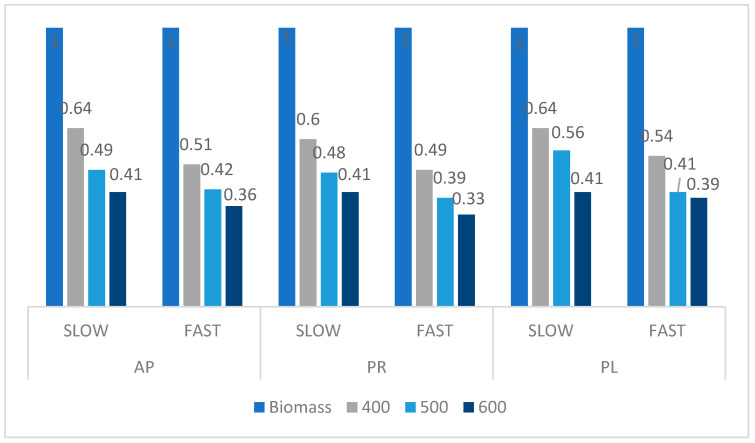
Comparison of the raw material/biochar energy ratios.

During the pyrolysis, the energy stored in chemical bonds is partially released during thermal degradation of the raw material and partially remains in the form of the solid residue (biochar) and volatile fraction. Therefore, the raw material/biochar energy ratio was calculated for each sample (based on HHVs) to assess the highest possible energy that could be generated during combustion of the solid product. The potential surplus energy from the combustion process can be recovered in cogeneration systems [52]. Various temperatures and heating rates were analyzed in these experiments to select the conditions to gain the highest pyrolysis efficiency and extract the maximum possible energy from the biomass, and the results are presented in Figure 7. The pruning wastes as a source of renewable energy were characterized by relatively high energy raw material/product energy ratios. Higher temperature of the pyrolysis resulted in a lower raw material/product energy ratio [53]. The lowest values were obtained for the pyrolysis processes performed at 600 °C (0.33 for PR, 0.36 for AP, and 0.39 for PL). The fast heating rate also resulted in lower energy ratios in comparison with SP. The energy ratio calculated for SP was comparable for all samples. For example, this parameter obtained for pyrolysis performed at 400 °C for AP was equal to 0.64, for PR 0.60, and for PL 0.64. The most beneficial (lowest) raw material/product energy ratio was detected for PR samples thermally treated in FP at 600 °C (0.33).

During pyrolysis, the volatile fraction (liquid and gas) provides an additional source of energy to the energy contained in the product [54,55]. The thermal treatment of biomass samples is an endothermic process, and the pyrolysis products are energetically valuable and allow for a positive energy balance of the process. In industrial process, the volatile fraction can be used as an energy source in endothermal processes. Pruning residue, as the raw material, has a relatively high moisture content, even up to 45%, therefore its drying requires a considerable amount of energy.

**Figure 8:**
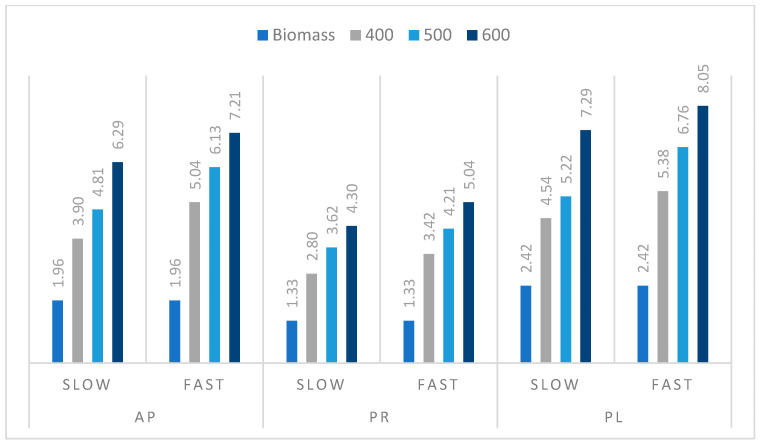
Raw material and biochar ash content (wt %).

As shown above, the carbon content in the biochars obtained from pruning residues was comparable (Figure 5), however, the ash content differed (1.33 wt % for PR, 1.96 wt % for PR, and 2.42 wt % for PL) (Figure 8). The inorganic components of the raw material did not change during the pyrolysis, and remained in the solid product. The degradation of the biomass, and thus the mass loss, was proportional to the concentration of organic fraction, which, in turn, depended on the content of inorganic compounds. When considering biochar as a fuel for industrial combustion, priority is given to the highest solid fraction yield, the lowest ash content, and the lowest energy ratio. In this case, the lowest ash content values were detected in the products obtained at 400 °C in SP for all samples (2.80 wt % for PR, 3.90 wt % for AP, and 4.54 wt % for PL). On the other hand, biochar also has the potential to be activated to become the active carbon with a high surface area [47]. The effect of the temperature of the slow pyrolysis process (300–750 °C) on the surface area of wood biochar samples was investigated, for example, by Ronsse et al. The highest surface area of the wood biochar sample in the slow pyrolysis process was obtained at the highest temperature of the process (600, 750 °C) [56].

**Figure 9:**
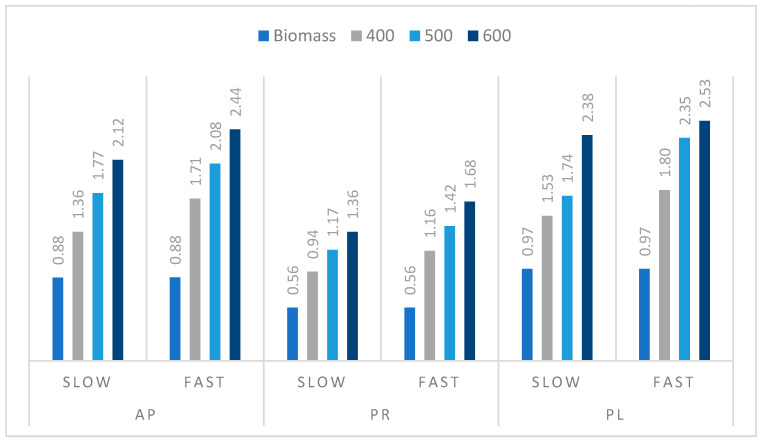
Raw material and biochar ash content per 1 MJ of energy ratio (g/MJ).

The HHV data and ash content determined for the selected prunings’ biochars obtained during our experiments (Figure 8) were used to calculate the amount of ash per 1 MJ of energy released during combustion (ash per 1 kg of biochar/HHV) (Figure 9). This is valuable information for the processes conducted at an industrial scale, since it reflects the amount of raw material needed for biochar production. The increasing temperature of the pyrolysis process resulted in higher ash energy ratios. The higher heating rate of the process also showed the same effect. The highest value of ash per 1 MJ energy was obtained for AP (2.44 wt %) and PL (2.53 wt %) biochars prepared by FP at 600 °C, and the lowest for the PR sample obtained at 400 °C during SP (0.94 wt %). Generally, SP provided biochar with lower values of this parameter in comparison with the process performed at a higher heating rate. The higher temperature of the pyrolysis process is related to an increase in the biochar ash content, which is a negative effect. However, the carbonization process improves other parameters of biochar, such as HHV, and thus the energy density, as well as biological stability.

## 4. Conclusions

Carbonization of tree pruning residues derived from orchards appears to be a convenient alternative approach to converting biowaste into a high-quality solid fuel with high HHV and high carbon content. The use of the pyrolysis process allows the generation of the energy necessary for the drying and thermal decomposition processes (through the generation of volatile fraction). The resulting solid product itself has a high energy potential, or can be further used, for example, as a material for the production of activated carbon [47]. The pyrolysis process of pruning residues produced biochar in the amount of 30–50% by mass of the starting raw biomass sample. Among the three tree species studied (apple, plum, and pear), the type of biomass was of secondary importance to the process conditions used, such as heating rate and final pyrolysis temperature. All raw materials used had similar HHV and carbon content; however, the ash content differed. The lowest ash content was obtained for PR, hence the biochar ash content per 1 MJ of energy ratio and the raw material/biochar energy ratio were also the lowest. The obtained results showed that a heating rate of 15 °C/min and a final temperature of 400 °C allowed us to obtain the highest biochar production efficiency. On the other hand, to obtain the biochar with the highest HHV and carbon content, fast pyrolysis and high final temperatures (100 °C/min and 600 °C) must be used. Under such conditions, the HHV of the biochars obtained exceeded 30 MJ/kg, and the carbon content was 80%.

## Figures and Tables

**Table 1:** Analysis of raw materials (pruning residues): apple trees (AP), pear trees (PR), and plum trees (PL).

	AP	PR	PL
Moisture, (%)	44.86	40.91	41.76
HHV ^1^, (MJ/kg)	22.39	23.60	24.90
Ash, (%)	1.96	1.33	2.42

^1^ Higher heating value.

**Table 2:** Pseudo-activation energy of orchard pruning pyrolysis carried out in the temperature range of 200–400 °C.

Sample	Temp. Range (°C)	E (kJ/mol)	x	R^2^
AP	193–387	28.97	0.097–0.690	0.9722
PR	199–399	32.08	0.085–0.709	0.9738
PL	209–391	37.00	0.078–0.713	0.9718

## Data Availability

Data sharing not applicable. Data sharing is not applicable to this article.
